# The Early Humor Survey (EHS): A reliable parent-report measure of humor development for 1- to 47-month-olds

**DOI:** 10.3758/s13428-021-01704-4

**Published:** 2021-11-18

**Authors:** Elena Hoicka, Burcu Soy Telli, Eloise Prouten, George Leckie, William J. Browne, Gina Mireault, Claire Fox

**Affiliations:** 1 School of Education, University of Bristol, Bristol, BS8 1JA UK; 2 Psychology, Nevşehir Haci Bektaş Veli University, Nevşehir, Turkey; 3 Department of Psychology, University of Sheffield, Sheffield, UK; 4 Behavioral Sciences, Northern Vermont University, Lyndonville, VT USA; 5 School of Childhood, Youth and Education Studies, Manchester Metropolitan University, Manchester, UK

**Keywords:** Humor, Joke, Preschool, Toddler, Survey

## Abstract

We created a 20-item parent-report measure of humor development from 1 to 47 months: the Early Humor Survey (EHS). We developed the EHS with Study 1 (*N* = 219) using exploratory factor analysis, demonstrating the EHS works with 1- to 47-month-olds with excellent reliability and a strong correlation with age, showing its developmental trajectory. We replicated the EHS with Study 2 (*N* = 587), revealing a one-factor structure, showing excellent reliability, and replicating a strong correlation with age. Study 3 (*N* = 84) found the EHS correlated with a humor experiment, however it no longer correlated once age was accounted for, suggesting low convergent validity. Subsamples of parents from Studies 2 and 3 showed excellent inter-observer reliability between both parents, and good longitudinal stability after 6 months. Combining participants from all studies, we found the EHS is reliable across countries (Australia, United Kingdom, United States), parent education levels, and children’s age groups. We charted expected humor development by age (in months), and the expected proportion of children who would appreciate each humor type by age (in months). Finally, we found no demographic differences (e.g., country: Australia, Canada, United Kingdom, United States; parents’ education) in humor when pooling all data. The EHS is a valuable tool that will allow researchers to understand how humor: (1) emerges; and (2) affects other aspects of life, e.g., making friends, coping with stress, and creativity. The EHS is helpful for parents, early years educators, and children’s media, as it systematically charts early humor development.

Humor is a human universal which is important to coping with stress, making friends, learning, being creative, and attracting mates (Bressler et al., 2006; Hoicka & Martin, 2016; Martin & Dobbin, 1988; Wanzer et al., 1996; Ziv, 1983). Yet there is little research about how humor emerges in the first place. Given humor’s universality and importance in so many aspects of children’s and adults’ lives, it is important that we develop tools to determine how humor first develops so that we can further understand not only the emergence of humor itself, but how humor may help young children function cognitively, socially, and in terms of mental health. The goal of the current set of studies is to determine: (1) the psychometric properties of a new parent-report measure of early humor development: the Early Humor Survey (EHS); (2) what types of humor are present in early development; and (3) the ages at which different types of humor emerge. The research presented here should also allow future experiments on early humor to be age-appropriate and empirically grounded, rather than based on researchers’ own assumptions about what might be humorous for young children.

While there is relatively little research focusing specifically on early humor development compared to other forms of play (e.g., pretending), experiments, observations, parent interviews, and parent surveys do give us some insights into when humor first develops, and what young children find funny at different ages. Humor is already present in the first year, with infants reported to appreciate different types of humor, including hide and reveal games (e.g., peekaboo), tickling, funny bodily actions, silly faces, strange voices and noises, showing hidden body parts, chasing, teasing, taboo topics, acting as something else, misusing objects, aggressive acts, and violating social rules (see Table 1) (Addyman & Addyman, 2013; Fernald & O’Neill, 1993; Hoicka & Akhtar, 2012; MacDonald & Silverman, 1978; Mireault et al., 2015; Mireault et al., 2014; Mireault, Poutre, et al., 2012; Reddy, 2001; Reddy & Mireault, 2015; Shultz, 1976; Sroufe & Wunsch, 1972). Indeed, infants were observed to appreciate and produce clowning as early as 3 months (Mireault, Poutre, et al., 2012), and parents have reported that some infants laugh as early as 1 month (Addyman & Addyman, 2013).

**Table 1:** Items; empirical sources for items; Spearman’s rho correlations between the final items and total scale (*r*); factor loadings for the exploratory factor analysis (EFA, Study 1), standardized regression weight means for the Bayesian confirmatory factor analysis (CFA, Study 2); and the ages (in months) at which 25, 50, and 75% of children are predicted to pass each item based on the logistic regressions of age on each item (see Study 5, Results). Where there are no ages under the percentiles, this indicates that by 47 months, fewer than that percentile (e.g., 50%) of children appreciated that type of humor. Numbers are in bold for the factor onto which the item loaded best. Items are ordered by age of emergence, based on the percentiles

**Item**	**Humor type**	**Question**	**Source**	***r***	**F1** **EFA**	**F2 EFA**	**F CFA**	**25%**	**50%**	**75%**
9	Hide & Reveal Games	Peekaboo/ hide & seek, including variations, e.g., hiding objects in bags and revealing them	(Addyman & Addyman, 2013; Fernald & O’Neill, 1993; Hoicka & Akhtar, 2012; MacDonald & Silverman, 1978; Shultz, 1976; Sroufe & Wunsch, 1972; Waters et al., 1975)	.41	– 0.10	**1.00**	.16	1	1	1
8	Tickling	Tickling, including variations, e.g., using objects to tickle, e.g., stick or feather	(Addyman & Addyman, 2013; Hoicka & Akhtar, 2012)	.33	– 0.15	**0.93**	.25	1	1	1
17	Funny faces	Pulling/making silly faces, e.g., scrunching up face	(Angeleri & Airenti, 2014; Hoicka & Akhtar, 2012; Loizou, 2004; Mireault, Poutre, et al., 2012; Reddy, 2001)	.46	0.04	**0.78**	.38	1	1	8
10	Bodily humor	Strange body movements, e.g., head through legs, kicking legs in air	(Addyman & Addyman, 2013; Hoicka & Akhtar, 2012; Loizou, 2004, 2005; Reddy, 2001; Sroufe & Wunsch, 1972; Tallant, 2017)	.59	0.30	**0.56**	.29	1	1	10
1	Funny voices	Making strange voices (not just strange noises)	(Sroufe & Wunsch, 1972; Tallant, 2015)	.42	0.13	**0.56**	.41	1	1	17
12	Chasing	Chasing, including variations, e.g., making toys chase each other	(Hoicka & Akhtar, 2012; Sroufe & Wunsch, 1972; White, 2014)	.68	0.41	**0.52**	.41	1	4	16
3	Misusing objects	Strange actions with objects, e.g., use wrong end of spoon, put cup on head	(Dubois et al., 1984; Hoicka, 2016a; Hoicka & Akhtar, 2012; Hoicka & Butcher, 2016; Hoicka & Gattis, 2008, 2012; Hoicka et al., 2008; Hoicka & Martin, 2016; Hoicka & Wang, 2011; Horgan, 1981; Loizou, 2004, 2005; Mireault et al., 2018; Mireault et al., 2015; Mireault et al., 2014; Mireault, Poutre, et al., 2012; Reddy, 2001; Sroufe & Wunsch, 1972)	.71	0.40	**0.64**	.42	1	8	17
19	Teasing	Teasing, e.g., offering an object and taking it away	(Howe et al., 2018; Reddy, 2001)	.62	0.31	**0.55**	.39	1	12	38
18	Showing hidden body parts	Showing normally hidden body parts, e.g., lifting shirt to reveal tummy; taking off clothes	(Reddy, 2001)	.62	0.38	**0.46**	.45	1	15	31
11	Scaring others	Scaring people, e.g., jumping out at them, or yelling	(unpublished corpus from Hoicka & Akhtar, 2012; see Appendix 39)	.68	0.44	**0.47**	.47	1	16	35
15	Acting like something else	Acting like something else, e.g., an animal, another person, etc.	(Addyman & Addyman, 2013; Reddy, 2001; Sroufe & Wunsch, 1972)	.68	**0.55**	0.36	.64	8	17	26
5	Taboo topics	Referring to gross things, e.g., poo, sneezing, smelly feet, etc.	(Addyman & Addyman, 2013; Hoicka & Akhtar, 2012; Howe et al., 2018; Reddy, 2001; Tallant, 2015)	.65	**0.71**	0.13	.67	15	23	31
6	Mislabeling	Mislabeling objects/events, e.g., calling a car a banana; could be in song, or intentionally giving you the wrong answer	(Hoicka & Akhtar, 2011, 2012; Horgan, 1981; Johnson & Mervis, 1997; Read et al., 2018)	.73	**0.91**	0.02	.62	22	29	36
2	Making fun	Making fun of others, e.g., calling someone a poopoohead	(Dubois et al., 1984; Hoicka & Akhtar, 2012)	.65	**0.75**	0.13	.63	24	30	37
7	Aggressive humor	Aggressive acts, e.g., spitting out water, throwing things, pushing people, etc.	(Esseily et al., 2016; Mireault, Poutre, et al., 2012; Reddy, 2001; White, 2014, 2017)	.63	**0.46**	0.39	.28	1	31	*–*
4	Playing with concepts	Saying strange things/mixing up concepts/nonsense (e.g., dinosaurs eat the wall; cats have 5 legs, dogs say moo), including nonsense variations of knock-knock/why did the chicken cross the road jokes	(Dubois et al., 1984; Hoicka & Akhtar, 2012; Hoicka et al., 2008; Hoicka & Gattis, 2012; Horgan, 1981; Johnson & Mervis, 1997; Read et al., 2018)	.73	**0.90**	0.01	.60	24	31	37
16	Nonsense words	Inventing words, e.g., schmoogly	(Hoicka & Akhtar, 2011, 2012; Hoicka et al., 2008; Hoicka & Gattis, 2012; Horgan, 1981; Johnson & Mervis, 1997; Loizou, 2004, 2005; Read et al., 2018)	.66	**0.86**	0.00	.56	28	35	42
13	Playing with social rules	Socially unacceptable situations, e.g., putting cat on dining table, saying naughty words, etc.	(Hoicka & Gattis, 2012; Hoicka et al., 2008; Hoicka & Wang, 2011; Mireault, Poutre, et al., 2012)	.64	**0.88**	0.02	.49	33	44	*–*
14	Tricks	Playing tricks on people, e.g., putting salt in the sugar bowl	(unpublished corpus from Hoicka & Akhtar, 2012; see Appendix A)	.32	**0.84**	– 0.26	.45	39	*–*	*–*
20	Puns	Making puns, that is, jokes where words have double meanings, e.g., Why are fish so smart? Because they live in schools	(Dubois et al., 1984; Johnson & Mervis, 1997; Loizou, 2005)	.52	**0.88**	– 0.06	.26	*–*	*–*	*–*
21	Funny noises (not included in final survey)	Making strange noises, e.g., raspberries, shrieks, sneeze sounds	(Addyman & Addyman, 2013; Loizou, 2004; Mireault et al., 2018; Mireault et al., 2014; Mireault, Poutre, et al., 2012; Reddy, 2001; Sroufe & Wunsch, 1972; White, 2014)	*NA*	*NA*	*NA*	*NA*	*NA*	*NA*	*NA*

One-year-olds’ humor is more established, continuing with earlier forms of humor, with the majority of 1-year-olds now engaging in tickling, chasing, and funny bodily actions, perhaps reflecting advances in motor development (Esseily et al., 2016; Hoicka, 2016a; Hoicka & Akhtar, 2012; Hoicka et al., 2008; Hoicka & Butcher, 2016; Hoicka & Gattis, 2008, 2012; Loizou, 2005). There has also been evidence of children producing basic puns, saying strange things as jokes, inventing words, and mislabeling objects from 1 year onwards (Horgan, 1981; Johnson & Mervis, 1997; Loizou, 2004). Two-year-olds’ humor reflects advances in cultural understanding, language development, and understanding of social rules, with most children now producing jokes involving misusing objects, saying strange things, inventing words, and addressing taboo topics (e.g., poo) (Hoicka & Akhtar, 2011, 2012; Hoicka & Martin, 2016). Finally, 3-year-olds’ humor reflects metalinguistic awareness with most children now capable of mislabeling (Hoicka & Akhtar, 2012).

The above demonstrates that humor is a complex, developing process in the first 4 years. While early humor research shows some overall patterns of humor development, the list of humor types covered is not exhaustive, and generally covers small age ranges (Addyman & Addyman, 2013; Dubois et al., 1984; Hoicka & Akhtar, 2012; Johnson & Mervis, 1997; Loizou, 2004, 2005; Mireault, Poutre, et al., 2012; Reddy, 2001; Sroufe & Wunsch, 1972). Additionally, while children responding to different types of humor within experiments gives us some empirical evidence about humor understanding at different ages (Esseily et al., 2016; Hoicka & Akhtar, 2011; Hoicka et al., 2017; Hoicka & Gattis, 2008; Hoicka & Martin, 2016; Hoicka & Wang, 2011; Mireault et al., 2014; Mireault et al., 2015; Mireault et al., 2018; Shultz, 1976; Sroufe & Wunsch, 1972; Waters et al., 1975), we do not know the extent to which these types of humor are enjoyed in everyday life. What is missing is: (1) a global measure of early humor development; and (2) a systematic taxonomy of humor development in the first years of life. We chose to focus on a global English-language survey as previous research found that parents reported instances of early humor from 25 different countries (Addyman & Addyman, 2013) suggesting early humor is universal. Given this, we wanted to create a survey that could be used in different English-speaking countries. This is important as the survey could theoretically benefit researchers in different countries, as well as allow international collaboration on research projects. Furthermore, in the current project, it would allow us to look for similarities and differences between countries.

This project is important for several reasons. First, we have no formal understanding of what types of humor will work at different ages. This is problematic for research, where we must decide to some extent on intuition as to which humorous acts to use in experiments, and how to code humor in observations. With a well-established humor taxonomy, based on hundreds of children, researchers could use this evidence base to guide their research design. Additionally, early years education around the world is based on play, with some frameworks explicitly including humor as a target (Australian Government Department of Education and Training, 2017; Best Start Expert Panel on Early Learning, 2007; Department for Education, 2017; Ohio Department of Education, 2012). However, with no formal understanding of when different types of humor develop, it could be difficult for early years educators to target effective humor for their students. This research could address that gap. This research would also be useful for parents who want to find new ways to play and joke with their children, as well as children’s media professionals who would like to target humor at specific ages of children.

A global measure of early humor development would also be incredibly useful in a research context. First, various theories suggest that humor development may be based on cognition, social development, language development, and social cognition (Freud, 1916; Hoicka, 2014, 2016b; Leekam, 1991; Loizou, 2005; McGhee, 1979; Reddy, 2001; Reddy & Mireault, 2015; Shultz, 1976). By having a global measure of early humor development, we can test these theories more rigorously, for instance, examining whether improvements in language, cognition, social skills, or social cognition predict advances in humor development. Second, as humor is important in coping with stress, making friends, learning, and being creative, in early life or later on (Hoicka & Martin, 2016; Martin & Dobbin, 1988; Wanzer et al., 1996; Ziv, 1983), a global measure of early humor development could allow us to predict what effect humor may have on these other areas of life in the early years.

In this study, we sought to create a parent-report measure of humor development from birth to 47 months. First, we generated a comprehensive list of potential types of humor appreciated by young children to include in a parent-report measure of early humor understanding: the EHS. We used exploratory factor analysis (EFA) in Study 1 (*N* = 219) to determine the EHS’s validity. Next, we used confirmatory factor analysis (CFA) in Study 2 (*N* = 587). Finally, participants in Study 3 (*N* = 84) completed a humor experiment in the lab, and their parents completed the EHS, to determine whether parent-reported humor and humor experiments correlate. In Study 4, we used a subsample of participants from Studies 2 and 3 to measure inter-observer reliability between parents, and 6-month longitudinal stability. Finally, in Study 5, we examined data from the first three studies together to determine whether the EHS had internal validity within different demographic groups (e.g., different countries: Australia, United Kingdom, United States; different levels of education); to determine whether there were differences between demographic groups; and we combined data from Studies 2 and 3 to determine the ages at which different forms of humor emerge; and to predict scores on the EHS by month.

## Study 1: Survey construction

We chose to examine humor from birth as infants have been observed to produce and appreciate clowning from 3 months (Mireault, Poutre, et al., 2012), and parents have reported that some infants laugh from 1 month (Addyman & Addyman, 2013). Therefore, to ensure we capture humor’s earliest emergence, as perceived by parents, we wanted the survey to be open to infants from birth. We chose 47 months as an end point to keep the range to the pre-school years, as compulsory schooling begins from 4 years (48 months) in the United Kingdom. The first author conducted a literature review of humor development across the 0 to 47-month age range. They searched for terms including “humor*” and “jok*” alongside terms such as “preschool*”; “toddler*” and “infan*” within abstracts on PsycInfo. They then read through the abstracts and downloaded papers which included participants within any part of the 0 to 47-month age range, and which clearly showed that one or more types of humor were observed or tested. They then included papers for which there was evidence of children in the 0 to 47-month age range producing specific types of humor (see Table 1). They also read through parents’ answers to an open-ended question about what types of humor young children produce accessed from the raw data of a previous short-form humor survey for parents of children from birth to 47 months (Hoicka & Akhtar, 2012). This was used to capture other types of humor not already captured in publications.

After generating a list of humor types appreciated in the 0 to 47-month age range, we next generated questions to ask about each type of humor, and generated specific joke tokens to better explain each type of humor. For instance, for item 3, we asked, “Strange actions with objects, e.g., use wrong end of spoon, put cup on head.” Therefore, the type of humor is “strange actions with objects” while example tokens we gave were, “use wrong end of spoon, put cup on head.” Other items were created in the same way (see Table 1 for experimental sources for items). This process led us to create 21 items that involved humor types that research found emerged from 3 months (clowning)(Mireault, Poutre, et al., 2012), to humor that is produced primarily by 3-year-olds (e.g., mislabeling) (Hoicka & Akhtar, 2012). We then tested the items on an initial pool of participants (DeVellis, 2017).

### Method

#### Participants

See Table 2 for power analysis. We obtained surveys for 219 children. See Table 3 for participant information. We do not report income statistics of samples with fewer than five participants in a country. Participants were recruited through Facebook advertizing, targeting parents of children 0–3 years in English-speaking countries; posts on lab and parenting Facebook pages; press releases; and Bounty packs in Sheffield, United Kingdom. There was no reward for participation.

**Table 2:** Power analyses for all analyses. N^req^ is the minimum number of participants required. N^act^ is the actual number of participants in the sample for each analysis. For Study 5, analyses were *a priori* for Child Age, Child Gender, Parent Age, and EHS Version, but *post hoc* for other demographic variables as we could not predict the breakdown ahead of time

**Analysis**	**Statistic**	**N**^**req**^	**N**^**act**^	**Source**
Study 1: Survey Construction	EFA	210	215	21 items; 10 participants per item (Tabachnick & Fidell, 2007)
Study 2: Survey Replication	CFA	200	587	Minimum 200 participants (Kline, 2011)
Study 3: Concurrent Validity	Correlation	84	84	Two-tailed medium correlation (*r* = 0.3, based on previous surveys) (Libertus & Landa, 2013; Winstanley & Gattis, 2013), with *α* = 0.05, power = 0.8 (Faul et al., 2007)
Study 4: Inter-observer Reliability	Correlation	29	39	Two-tailed large correlation (*r* = 0.5, based on previous surveys) (Putnam et al., 2006), with *α* = 0.05, power = 0.8 (Faul et al., 2007)
Study 4: Longitudinal Stability	Correlation	29	214	Two-tailed large correlation (*r* = 0.5, based on previous surveys) (Putnam et al., 2006), with *α* = 0.05, power = 0.8 (Faul et al., 2007)
Study 5: EHS Version	Differential Item Functioning	200/ group	≥214/ group	Small effect size (based on simulations, corrections for multiple testing) (Belzak, 2020)
Study 5: Age, Parent Education	Differential Item Functioning	400	873-886	Small effect size (based on simulations, corrections for multiple testing) (Belzak, 2020)
Study 5: Parent Education (Degree, No Degree); Country (UK, US)	Differential Item Functioning	100/ group	≥112/ group	Medium effect size (based on simulations, no corrections for multiple testing) (Belzak, 2020)
Study 5: Country (UK, Australia)	Differential Item Functioning	25/ group	≥30/ group	Large effect size (based on simulations, no corrections for multiple testing) (Belzak, 2020)
Study 5: Child Age, Parent Education, Country	KR(20)	30	30-674	Based on simulations, when first eigenvalue above 6 (Yurdugül, 2008)
Study 5: Child Age	Linear regression	787	873-886	Small effect size (Cohen’s *f* = 0.1) with *α* = 0.05, power = 0.8 (Faul et al., 2007)
Study 5: Child Gender	ANCOVA	394/groups	≥434/ group	Small effect size (Cohen’s *f* = 0.1) with *α* = 0.05, power = 0.8 (Faul et al., 2007)
Study 5: Childcare Hours, Income (UK)	Linear regression	259	434-605	Small to medium effect size (Cohen’s *f* = 0.175) with *α* = 0.05, power = 0.8 (Faul et al., 2007)
Study 5: Parent Education (Degree, No Degree), Siblings, Multilingualism	ANCOVA	130/ group	≥ 142/ group	Small to medium effect size (Cohen’s *f* = 0.175) with *α* = 0.05, power = 0.8 (Faul et al., 2007)
Study 5: Income (US)	Linear regression	77	90	Medium to large effect size (Cohen’s *f* = 0.325) with *α* = 0.05, power = 0.8 (Faul et al., 2007)
Study 5: Parent Gender	ANCOVA	39/group	≥51/ group	Medium to large effect size (Cohen’s *f* = 0.325) with *α* = 0.05, power = 0.8 (Faul et al., 2007)
Study 5: Country (Australia, Canada, UK, US)	ANOVA, and ANCOVA	13/group	≥16/ group	Large effect size (Cohen’s *f* = 0.40) with *α* = 0.05, power = 0.8 (Faul et al., 2007)

**Table 3:** Participant information

	**Study 1**	**Study 2**	**Study 3**
**No. of children/ parents reporting**	219	587	84
**Children’s age:**			
*Mean* (months; days) *Range* *SD*	19;24 0;7–47;14 12;14	27;9 1;28–47;28 11;3	23;27 1;19–46,6 13;7
**Children’s gender:**			
Female	86 (39%)	279 (48%)	43 (51%)
Male	132 (60%)	307 (52%)	41 (49%)
Not reported	1 (0.5%)	1 (0.2%)	0 (0%)
Children’s ethnicity*:			
Of Color	27 (12%)	51 (9%)	5 (6%)
White	186 (85%)	531 (90%)	78 (93%)
Not reported	6 (3%)	5 (1%)	1 (1%)
**Country:**			
Australia	27 (12%)	3 (0.5%)	0 (0%)
Canada	10 (5%)	7 (1%)	0 (0%)
United Kingdom	111 (51%)	481 (82%)	84 (100%)
United States of America	47 (21%)	66 (11%)	0 (0%)
Other	22 (10%)	24 (4%)	0 (0%)
Not reported	2 (1%)	6 (1%)	0 (0%)
**Child’s language**			
English only	167 (76%)	466 (79%)	63 (75%)
English and another language(s)	43 (20%)	85 (14%)	13 (15%)
Other language only (monolingual)	0	2 (0.3%)	0 (0%)
Other languages only (multilingual)	0	2 (0.3%)	0 (0%)
English, parents did not report whether children were exposed to another language	0	27 (5%)	8 (10%)
Not reported	9 (4%)	4 (1%)	0 (0%)
**Sibling(s)**			
Yes	75 (34%)	278 (47%)	36 (43%)
No	141 (64%)	281 (48%)	40 (48%)
Not reported	3 (1%)	28 (5%)	9 (11%)
**Childcare** (hours)			
*Mean*	*N/A*	17.22	12.67
*Range*		0–75	0–50
*SD*		14.56	12.69
Not reported	218	60	7
**Reporting parents’ age (years)**			
*Mean*	33.22	33.75	34.20
*Range*	18–46	18–62	22–43
*SD*	4.60	4.99	4.20
Not reported	1	33	7
**Reporting Parents’ Gender**			
Female	209	514	75
	(95%)	(88%)	(89%)
Male	8	41	2
	(4%)	(7%)	(2%)
Not reported	2	32	7
	(1%)	(5%)	(8%)
**Reporting parents’**			
Ethnicity*:	14	43	3
Of Color	(6%) 201	(7%) 512	(4%) 73
White	(92%) 4	(87%) 32	(87%) 8
Not reported	(2%)	(5%)	(10%)
**Reporting parents’ Education**			
High School	26	66	15
	(12%)	(11%)	(18%)
Community College	9	27	0
	(4%)	(5%)	(0%)
Undergraduate Degree	64	212	37
	(29%)	(36%)	(44%)
Postgraduate Degree	116	272	32
	(53%)	(46%)	(38%)
Not reported	3	10	0
	(1%)	(2%)	(0%)

*See Appendix Table 6 for a more detailed breakdown of ethnicity, as well as information on household income, and recruitment.

Ethical approval was obtained from the Psychology Department at the University of Sheffield for the projects, “Using parent reports to learn about early humor, pretending, deception, creativity, social cognition, actions, and language”, Reference Number 003095, and “The relationship between humour development and social cognition from 3 months to 47 months: A lab study”, Reference Number 013845. Parents who completed the survey on www.babylovesscience.com ticked boxes online to indicate their consent for the survey. Parents who completed the survey in the lab ticked boxes and signed a paper consent form. We report how we determined our sample size, all data exclusions (if any), all manipulations, and all measures in the study.

#### Measure

##### Preliminary Early Humor Survey

The initial survey consisted of 24 basic questions with contingent follow-up questions (see Appendix Table 7). The first three questions were more general, e.g., “Does your child appreciate humor? (It could be verbal or physical, e.g., silly faces).” We also asked if children produced humor or laughed, following Addyman and Addyman (2013), with parents able to choose “Yes” or “No.” If parents answered “Yes” questions were followed up, e.g., “When was the last time your child appreciated humor?” We asked about time to determine how often children appreciated or produced humor. If parents answered “No” there were no follow-up questions. The next 21 questions were about specific types of humor (see Table 1 and Appendix Table 7). Each question was headed e.g., “Strange actions with objects, e.g., use wrong end of spoon, put cup on head.” followed with questions, “Has your child ever seen anyone make this type of joke?”; “Has your child ever found it funny when others produced this type of joke?”; “Has your child ever tried to make this type of joke?”; “Has your child ever correctly copied this type of joke from others?”; and “Has your child ever invented this type of joke correctly him/herself?” We divided questions in this way to not only distinguish humor appreciation and production, but to also distinguish copying and inventing humor, with the latter appearing later in development according to previous research (Hoicka & Akhtar, 2012). These questions were also contingent. For instance, we only asked if children found the type of joke funny when others produced it if they had actually seen it; and we only asked if children copied or invented a joke type if their child had tried to produce it. Furthermore, if parents said at the beginning of the survey that their child had never produced jokes, we only asked about humor appreciation. We set the survey up in this manner to make it shorter where possible. For instance, we would not expect 3-month-olds to have attempted to produce the vast majority of jokes, so did not want to waste parents’ time asking details about jokes their children had not attempted to produce. Participants completed the survey on their own laptop through the website www.babylovesscience.com.

### Results

**Fig. 1:**
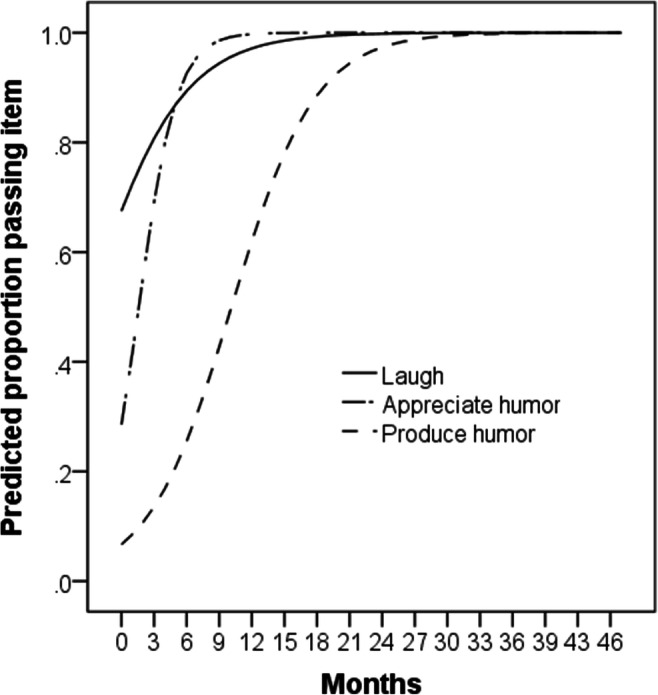
Predicted age curves for laughter, humor appreciation, and humor production

To get a general understanding of children’s humor appreciation and production, we analyzed whether children laughed, appreciated humor, or produced humor in general. We distinguished laughter and humor appreciation as children may laugh without there being any clear joke; or they may simply smile at jokes when appreciating them without laughing. Out of 219 children, 209 (95.43%) were reported to laugh, 207 (94.52%) were reported to appreciate humor, and 153 (69.86%) were reported to produce humor. For each of these three items, we ran binary logistic regressions for each item score on age (in months) as the independent variable. Age was a significant positive predictor of each of the above behaviors, all *N* = 219, *Wald* > 9.04, *β* > .232, *p* < .004. We then plotted the predicted proportion of children displaying each behavior, by age (see Fig. 1). More than 50% of children were predicted to laugh by 0 months, 75% by 2 months and 97.5% by 13 months. More than 25% of children were predicted to appreciate humor by 0 months, 50% by 2 months, 75% by 4 months, and 97.5% by 8 months. More than 25% percent of children were predicted to produce humor by 6 months, 50% by 11 months, 75% by 15 months, and 97.5% by 25 months.

We also examined how prevalent humor is in everyday life by asking parents who reported humor appreciation or production to tell us when the last time their child appreciated or produced humor was. *N* = 184 parents reported on when children last appreciated humor; Quartile (Q)1 = 1 hour, Q2 = 2 hours, and Q3 = 4 hours, range = 0 minutes – 1 week. There was no correlation between children’s age (in days) and how long ago they appreciated humor, Spearman’s rho *r* = .025, *p* = .737. Therefore, humor appreciation is very common, and not age-related, with at least half of children in the sample having appreciated humor in the last 2 hours; and this is a conservative estimate as some parents may have answered after children had gone to bed. The other 23 parents who reported humor appreciation either did not answer this question, or did not answer it according to our instructions, e.g., “Yesterday” which could not be collapsed into hours or days since we did not know what time it was when they answered the question nor the time the event took place; or they gave an anecdote without referring to time.

A total of 135 parents reported on when children last produced humor; Q1 = 1 hour, Q2 = 3 hours, and Q3 = 12 hours, range = 10 minutes – 3 weeks. There was no correlation between children’s age (in days) and how long ago they produced humor, Spearman’s rho *r* = – .147, *p* = .089. Therefore, humor production is also very common, with at least half of children in the sample having produced humor in the last 3 hours. Once children produce humor, they produce it often, with no further developmental changes. The other 20 parents who reported humor production either did not answer this question, or did not answer it according to our instructions. A Wilcoxon signed-ranks test of the 135 children who both appreciated and produced humor found they appreciated humor significantly more recently than they produced humor, *Z* = 5.66, *r* = .49, *p* < .001, suggesting humor appreciation is more frequent than humor production.

We next looked at the different types of humor. We found that for humor appreciation, copying jokes, and inventing jokes, scores were always 0 out of 21 (all items summed) for children under 1 month, but sometimes higher for children from 1 month. Therefore, we removed children under 1 month (*N* = 4) from the analyses, and retained children from 1 month onwards. Total copying jokes and inventing jokes scores were positively skewed, so we used non-parametric tests. A Friedman test found a significant difference across humor appreciation, copying jokes, and inventing jokes, *N =* 215*, χ*^*2*^(2) = 297.06, *p* < .001. Follow-up Wilcoxon signed-ranks tests found children had significantly higher humor appreciation (median = 11) than both copying joke (median = 6) and inventing joke scores (median = 4); and children had significantly higher copying joke than inventing joke scores, all *N =* 215*, Z* > 4.09, *r* > .28, *p* < .001. Spearman’s rho correlations found all three constructs were very strongly correlated with each other, all *N =* 215*, r* > 0.819, *p* < .001.

Due to the very high correlations between the number of types of humor children appreciated, copied, and invented, we collapsed questions for each humor type based on whether children had appreciated *or* copied *or* invented each type of humor to look at the questions as a single developmental measure. While these measures may still vary in terms of mean scores, analyzing multiple (nearly) collinear items for individual differences seemed redundant, and made the EHS unnecessarily long. We used Spearman’s rho correlations with age to determine whether all items increased with age as we sought to develop a survey that reflects development. Twenty of the items showed a positive increase with age, (all Spearman’s rho*, r* > .198, *p* < .004), suggesting they were all appropriate for inclusion in the survey, but not the item, “Making strange noises, e.g., raspberries, shrieks, sneeze sounds.” (*r* = .106, *p* = .120). This may be because it was present for most of the sample (*N* = 193/215, or 89.8%) so may already have been at a ceiling level early on. Therefore, this item was cut as it did not reflect humor development in this age range, even though it was a common type of humor. None of the remaining 20 of the collapsed items for Sample 1 were collinear (all Spearman’s rho, *r* < .692, *p* > .001), so all remaining items were retained.

We next examined whether each collapsed item correlated with the total humor score above *r* > .3, *p* < .05 (Pedhazur & Schmelkin, 1991). All items positively correlated with the total humor score (all 20 items Spearman’s rho, *r* > .318, *p* < .001, see Table 1). The Kuder–Richardson coefficient of reliability for binary items (analogous to Cronbach’s alpha for multipoint scales) indicated that the scale validity for the remaining 20 items was excellent, Kuder–Richardson Formula 20 (*KR20*) = 0.91.

Next, we examined whether the total humor score correlated with age (in days), since our purpose was to create a survey that tracks development. In our sample, the age distribution was positively skewed, therefore we used a Spearman’s rho correlation, which showed a very strong correlation between the total humor score and age, *N* = 215, *r* = .824, *p* < .001.

**Fig. 2:**
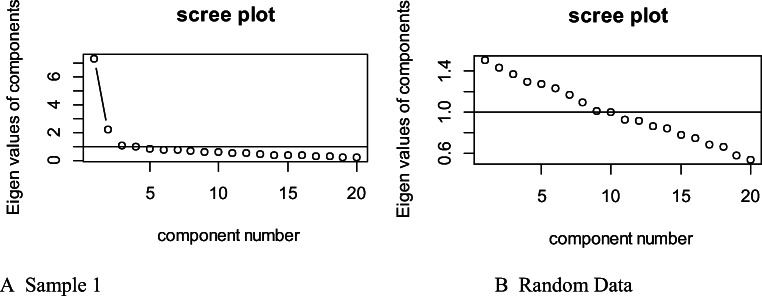
Scree plot for (**A**) Sample 1, and (**B**) random data, for parallel analysis

Finally, we performed an EFA for binary items in R (Starkweather, 2014) using the psych package (Revelle, 2014). Two factors loaded at eigenvalues above 1, and all other factors were around 1 or lower. Using parallel analysis, we see that both factors are above what would be expected by chance (see Fig. 2). This suggests a two-factor model. We therefore ran an EFA for binary items with two factors with oblimin rotation to allow factors to correlate. This accounted for 67% of the variance. Table 1 shows the factor loadings for each item. Sixteen of the 20 items loaded onto Factor 1 at a weighting of .30 or more, which accounted for 39% of the variance of the model. Items that loaded more strongly onto Factor 1 were those that were passed at a later age (see Table 1), and tended to reflect representational forms of humor, including verbal humor (e.g., mislabeling, puns), pretense (acting like something else), and understanding mental representations (e.g., making fun, tricks). Twelve items loaded onto Factor 2, at a weighting of .36 or more, which accounted for 28% of the variance of the model. Items that loaded more strongly onto Factor 2 were those that were passed at an earlier age (see Table 1), and tended to reflect physical forms of humor including misusing objects, hide and reveal games, and funny faces. Therefore, the two-factor structure picked up on age, which we aimed to capture in the EHS, as well as representational versus physical forms of humor. While most items loaded onto both factors, we put in bold the factor that each item loaded onto best, with ten items loading best onto each factor. Additionally, both factors were strongly correlated Spearman’s *r* = .60, *p* < .001 (Spearman’s *R* used as Factor 1 was positively skewed, and Factor 2 was negatively skewed). Internal reliability was good for both Factor 1, *KR(20)* = 0.84, and Factor 2, *KR(20)* = 0.71.

### Discussion

Study 1 found that 20 of the 21 EHS items increased with age, correlated with the total score, and showed good internal reliability. An EFA suggested a two-factor structure, with factors linking to age and representation, including language. Study 2 examined whether we could replicate internal reliability and the factor structure in a separate sample of participants.

## Study 2: Replication

### Method

#### Participants

See Table 2 for the power analysis. There were 587 children in Study 2. While only 200 children were required for replication, we aimed to recruit at least 550 so that all three studies would add up to at least 787 for key demographics analyses in Study 5 (e.g., child age, gender, see Table 2). Participants were recruited as in Study 1. All participants completed a demographics survey (see Table 2). There was no reward for participation, unless participants repeated the survey 6 months later, or the child’s other parent also completed the survey (see Study 4).

#### Measure

##### EHS

The final EHS was a much more streamlined version of the survey, for which there were only 20 questions based on the 20 types of humor. The instructions were, “For the following, tick Yes if your child finds it funny when others make this joke type and/or makes this joke type him/herself to be funny.” followed by the 20 types of humor (see Table 1 and Appendix Table 7 for the final 20 items). This was to reduce the time taken for the survey, given that the previous survey contained much redundancy.

### Results

We first performed a CFA using two factors, which were allowed to correlate. We performed this via a Bayesian structural equation model (SEM) implemented in AMOS 26 as items had binary values (Arbuckle, 2018). We used modification indices above 4 to determine which error terms correlated in order to improve model fit. We used modification indices to determine which representational item error terms correlated within Factor 1, and which non-representational item error terms correlated within Factor 2, but did not correlate items between factors. We correlated the following error terms for each item, within each factor, to improve model fit: 1 with 3, 8, 11, 12, 17, and 18; 2 with 4, 5, 7 and 15; 3 with 9, 11, 12, and 19; 4 with 6 and 16; 5 with 16; 6 with 13, 16, and; 7 with 13 and 16; 8 with 9, 10, 17, and 19; 10 with 11; 13 with 14, 15, and 20 ; 14 with 20 ; and 18 with 19. We used the Random Walk tuning parameter set to 0.4. Convergence was set to 1.1 (Gelman et al., 2013), and the model reached convergence. The model was not adequate, with posterior predictive *p* value (PPP) = .01, and deviance information criteria (DIC) = 348.10.

Since we could not fit a two-factor model, and both factors in Study 1 were highly correlated, we next tried to fit a one-factor model. We used modification indices to determine which error terms correlated in order to improve model fit. However, we only included these correlations if there was a logical reason that items would overlap, e.g., both items involved verbal humor (e.g., mislabeling, puns), or both items involved potentially making others uncomfortable (e.g., teasing, aggressive humor). Using this approach, we correlated the error terms of the following items. Sensory-based (e.g., sounds, physical) humor error term correlations included: 1 with 8, 9 and 17; 3 with 7, 9, 10, and 12; 7 with 10, 11, 18, and 19; 8 with 9, 10, 11, 12, 15, and 17; 9 with 10, 11, 12, 17, and 19; 10 with 12; 11 with 12; 12 with 17; and 18 with 19. Verbal humor error term correlations included: 2 with 4, 5, 6, and 16; 6 with 16; 4 with 5, 6, 16, and 20; 6 with 16; and 16 with 20. Error term correlations for humor which breaks social rules included: 2 and 13; and 7 and 13. Error term correlations for humor which might make others uncomfortable included: 5 with 19; 8 with 13; and 13 with 14. We used the Random Walk tuning parameter set to 0.4. Convergence was set to 1.1 (Gelman et al., 2013), and the model reached convergence. The model was adequate, with PPP = .13, and DIC = 330.15. The standardized regression weight means show that the 20 predicted items loaded onto the one factor at values of .16 or higher (see Table 1). All items had their standardized 95% credible intervals starting above 0, suggesting effect sizes were consistently positive.

Internal reliability on the 20 items of the EHS was very good, *KR20* = 0.86. This suggests the 20 items form a coherent scale to capture early humor. Next, we examined whether the total EHS score correlated with age (in days). EHS scores were negatively skewed, therefore we used a Spearman’s rho correlation, which showed a very strong correlation, *N* = 587, *r* = .712, *p* < .001.

### Discussion

Study 2 replicated Study 1’s finding that the EHS had very good internal reliability with a separate sample of participants. Additionally, CFAs suggested a one-factor structure was more appropriate than a two-factor structure. Study 2 also found the EHS correlated strongly with age. Study 3 sought to find convergent validity between the EHS and a researcher-led humor experiment.

## Study 3: Convergent validity

### Method

#### Participants

See Table 2 for the power analysis. There were 84 children in Study 3. Participants were recruited through Bounty packs within Sheffield, United Kingdom, press releases, and Facebook advertizing within Sheffield, United Kingdom; and their demographic details can be found in Table 3. This sample was selective as additional children were not included because children did not want to participate (e.g., stating they did not want to play the game, or e.g., crying for younger children; *N* = 24), experimental error (*N* = 4), the EHS was not submitted (*N* = 4), technical problems with the videos (*N* = 3), parents who showed children what to do (*N* = 2), or because they were distracted (by food and sibling, *N* = 1). Eighteen of the children who did not participate still had completed surveys, which we used in Study 2. We examined whether there were any age or gender differences between our final sample (*N* = 84) and the children who did not want to participate (*N* = 24 for gender, *N* = 23 for age, as one parent did not report it). An independent-samples *t* test for age violated Levene’s test for equality of variance, *F* = 6.40, *p* = .013. When equal variance was not assumed, there was no difference in mean age between the children who participated (*M* = 726.58 days, *SD* = 402.31) and those who did not (*M* = 740.91, *SD* = 295.05), *t*(46.88) = 0.19, *p* = .850. A Mann–Whitney *U* test for gender found no difference between children who participated (43 female, 41 male) and those who did not (eight female, 16 male), Mann–Whitney *U* = 1273.50, *Z* = 1.54, *p* = .124. Only six parents submitted the EHS of the children who chose not to participate. We ran a linear regression on EHS scores as the dependent variable, and age as the independent variable on children who completed the experiment and those who chose not to in order to obtain unstandardized residuals of EHS scores, controlling for age. The unstandardized residuals, controlling for age, for children who chose not to participate (*M* = 0.96, *SD* = 3.83) were higher than for those children who did choose to participate (*M* = – 0.07, *SD* = 3.09). Therefore, we do not have evidence that the children who chose not to participate understood humor less well than children who did, although with such a small sample, one must be cautious with these descriptive statistics. Children received a book for participating.

#### Measures

##### EHS

Same as Study 2.

##### Humor Appreciation Task

An experimenter modeled 21 jokes and 21 control acts across the study (see Appendix Table 8 for acts and materials). The experimenter always modeled a block of four or five control acts first (e.g., the experimenter held a toy horse and said, “The horse goes neigh! Neigh!”) which matched the content of the jokes (e.g., the experimenter held a toy horse and said, “The horse goes Quack! Quack Quack!”), followed by a block of four or five jokes. This was to (1) ground children in what normal versions of these acts look like to contrast with the jokes, and make the jokes more entertaining, and (2) use as a control condition to ensure children appreciated the jokes as jokes. For each control act or joke, the experimenter modeled the act while smiling, and gave an ambiguous laugh which could be interpreted as joy or humor, to keep the acts naturalistic, while maintaining experimental control between conditions (Hoicka & Akhtar, 2011). After each act, the experimenter said, “Now you joke!” (humor condition) or “Now you try!” (control condition). Jokes and control acts were ordered based on the number of children who were reported to appreciate each joke type in Sample 1, starting with the joke type that was reported to be appreciated the most, and ending with the joke type that was reported to be appreciated the least. If children did not laugh, or imitate while smiling or laughing, at all during a joke block, the test was ended early. This was because our study included children from a wide age range, from 1 to 47 months. Therefore, we did not expect younger children (e.g., 6-month-olds) to have any understanding of later types of humor (e.g., puns). Thus, we used this rule to end the task early when children clearly could not proceed, so as to avoid any stress for participants. We did not use smiling alone as a marker of humor appreciation as it is not possible to observe at all times while running an experiment. In contrast, the experimenter could always hear laughter. Parents were involved in some of the jokes and control acts as the “butt” of the joke.

##### Coding

Each joke and control act was coded from video as 1 if children laughed when the experimenter performed the act, or if the child imitated the act while smiling or laughing (Hoicka, 2016a; Hoicka & Akhtar, 2011, 2012; Hoicka et al., 2008; Loizou, 2005; Mireault et al., 2015; Mireault, Poutre, et al., 2012; Sroufe & Wunsch, 1972). Children scored 0 if they did not laugh while the experimenter performed the act, and did not imitate the act while smiling or laughing. This was to capture whether children either appreciated or produced each type of humor, in line with the EHS. However, if children were simply joyful and/or imitative, they might score high on the humor task, which is why we also coded the control trials in the same way. If children failed to laugh when the experimenter performed the joke, or imitate the joke while smiling or laughing, for an entire block of jokes, coding was stopped, to be in line with the stop rule of the experiment. Total humor appreciation/ production scores were obtained by summing all humor trials. The control joy/imitation scores were obtained by summing all the control trials. However, we did not include the “strange noises” joke and control acts as the item was not retained in the EHS. A second coder coded 17 (20%) of the videos. Agreement was excellent for humor scores, intra-class correlation (ICC) = 0.998, and for control scores, ICC = 0.995.

### Results

**Fig. 3:**
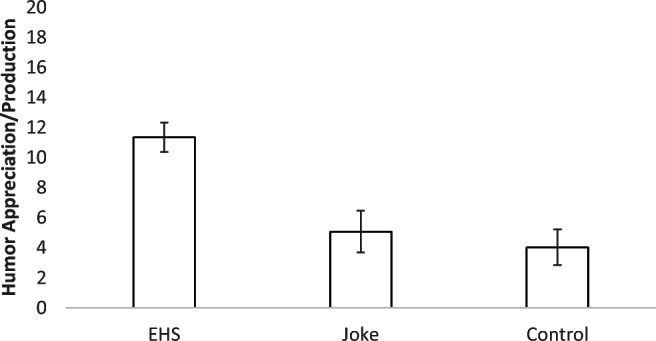
Mean humor appreciation/production scores for the EHS and joke and control trials in the humor experiment for Study 3. *Error bars* represent 95% confidence intervals (*N* = 84)

We first examined whether our lab task captured humor understanding by comparing children’s responses on the joke and control trials (see Fig. 3 for means and confidence intervals). A paired-samples *t* test found children laughed at the experimenter’s actions, or copied the experimenter’s actions while laughing or smiling, significantly more often on the joke than control trials, *t*(83) = 4.13, *p* < .001, *Cohen’s d* = 0.45. This suggests the experiment was effective at capturing humor on a group level with a medium effect size.

See Fig. 3 for the mean and confidence interval for the EHS score. Scale validity for the 20 EHS items was again very good, *N* = 84, *KR20* = 0.88. All 20 joke trials on the lab task correlated with the total joke lab scores (all Spearman’s rho *r* > .383, *p* < .001, see Table 4). Internal reliability across the humor lab trials was excellent, *N* = 84, *KR20* = 0.96. To control for general joyfulness/ copying, we subtracted total control scores from total joke scores in the experiment. The difference score was positively skewed. A Spearman’s rho correlation found a small to medium positive correlation between the difference scores and the EHS, *r* = .273, *p* = .001. We then examined whether the scores still correlated when age (in days) was controlled for. There was no correlation between the difference score and the EHS when age (in days) was partialed out, *r’* = – .062, *p* = .578.

**Table 4:** Spearman’s rho correlations between humor lab tasks and the total humor lab score (i.e., total number of trials for which children laughed at a joke, or copied a joke while smiling or laughing). *p < .05

**Task**	***r lab***
Hide & Reveal Games	.758*
Tickling	.760*
Funny faces	.520*
Bodily humor	.730*
Misusing objects	.702*
Chasing	.775*
Funny voices	.811*
Acting like something else	.778*
Teasing	.705*
Scaring others	.881*
Showing body parts	.758*
Taboo topics	.788*
Mislabeling	.637*
Aggressive humor	.817*
Making fun	.754*
Playing with concepts	.691*
Nonsense words	.461*
Playing with social rules	.713*
Tricks	.703*
Puns	.384*

### Discussion

Study 3 found that, as a group, children showed humor appreciation more on the joke trials than the control trials. Additionally, the difference scores of children’s humor response to joke and control trials correlated with the EHS. However, this correlation disappeared when age was controlled for. Therefore, the EHS did not show good convergent validity with a researcher-led experiment. Study 4 sought to determine whether we could demonstrate inter-observer reliability from both parents; and whether parents reported consistent EHS scores over a 6-month interval.

## Study 4: Inter-observer Reliability and Longitudinal Stability

### Method

#### Participants

See Table 2 for power analyses. Parents from Study 2 were invited to have their child’s other parent complete the survey as well. Reliability between parents was run for a subsample of participants from Study 2 (i.e., those who chose to participate, *N* = 39; 22 female children, 17 male; mean child age = 30 months, 11 days; *SD* = 10 months, 0 days; range = 1 month, 28 days to 45 months, 15 days). Parents from Studies 2 and 3 were invited to repeat the survey 6 months later. Six-month longitudinal stability was run for a subsample of participants from Studies 2 and 3 (i.e., those who chose to participate, *N* = 214; 99 male, 115 female; Time 1 mean = 26 months, 5 days; *SD* = 11 months, 6 days; range = 3 months, 17 days to 47 months, 14 days). While many more participants repeated the survey than required, we decided to analyze all participants’ data. Up to £2 was donated to charity (e.g., UNICEF), or a £5 Amazon voucher (or equivalent value in other countries) was donated to the parents, for each survey that was repeated, or for which a second parent completed the survey.

#### Measure

##### EHS

Same as Study 2. This was repeated by the other parent (inter-observer reliability), or by the same parent 6 months later (longitudinal stability).

### Results

#### Inter-observer reliability

On average, when both parents (*N* = 39 children) completed the surveys, they completed them 4.7 days apart (*SD* = 5.1 days; range = 0–16 days). Scale validity for the 20 items was very good for all parents together, *N* = 78, *KR20* = 0.86. Total scores on the EHS for the first set of parents were negatively skewed, therefore we used Spearman’s rho. Total scores on the EHS for parents 1 and 2 were very strongly correlated (Spearman’s rho *r* = .78, *p* < .001). A partial correlation, controlling for child age, found a very large correlation between parents’ surveys (*r’* = .72, *p* < .001). Using a potentially more robust measure, the EHS showed excellent reliability between parents, ICC using 1-way random effects = .92, *p* < .001.

#### Longitudinal stability

A subsample of parents from Studies 2 and 3 (*N* = 214) completed the EHS on average 6 months and 3 days after first completing it (*SD* = 12 days; range = 5 months, 0 days to 7 months, 0 days). Scale validity for the 20 items was very good at Time 1, *KR20* = 0.86, and Time 2, *KR20* = 0.83. EHS scores at Times 1 and 2 were very strongly correlated, Pearson’s *r* = .765, *p* < .001. A partial correlation, controlling for age at Times 1 and 2, found a significant positive large correlation between the EHS at Times 1 and 2 (*r’* = .551, *p* < .001).

### Discussion

Study 4 found that the EHS has excellent inter-observer reliability between parents, and good longitudinal stability after 6 months. Study 5 sought to determine whether the EHS could be used across different demographic groups, and to implement the EHS as a research tool to examine demographic differences.

## Study 5: Demographics

### Method

#### Participants

See Table 2 for power analyses. For the age analyses, we pooled participants from Studies 2 and 3, where children were at least 1 month old (*N* = 671). For the reliability and demographic differences analyses we pooled participants from Studies 1–3, where children were at least 1 month old (*N* = 886).

#### Measures

##### EHS

Same as Studies 1 and 2. We also measured demographics including age, child gender, parent gender, parent education, household income (United Kingdom or United States), country (Australia, Canada, United Kingdom, United States), multilingualism, siblings, and childcare hours (see Table 2).

### Results

Analyses to look for differences in EHS Version (preliminary, final), Child Age, Child Gender, and Parent Age could be planned for small effect sizes *a priori* as we aimed for minimum sample sizes per EHS Version, Child Age and Parent Age are continuous, and Child Gender was expected to be fairly evenly split. However, our power analyses for other demographic variables had to be done *post hoc* as (1) these demographic questions were optional for ethical reasons, e.g., not everyone feels comfortable reporting their income, therefore we could not predict how many participants would answer these questions; and (2) we could not predict the make-up of the participants for the other demographic variables, e.g., Parent Education. Therefore, Table 2 shows the *a priori* power analyses for EHS Version, Child Age, Child Gender; and Parent Age, and the *post hoc* power analyses for the other demographic variables.

#### Reliability across different demographic groups

We used differential item functioning (DIF) to determine whether item responses loaded onto the EHS differed by EHS Version or key demographic variables. This was done with logistic regression, with each EHS item as the dependent variable, the total EHS score as the independent variable in Step 1, and both the demographic variable, and the interaction of the demographic variable and total EHS score in Step 2. If there was a significant difference between the models in Steps 1 and 2, we looked at the difference in variance explained by each model (the Zumbo–Thomas effect size). If the Zumbo–Thomas effect size was above .13, this would indicate that people in different demographic groups responded differently to the item (Zumbo, 1999). EHS scores were negatively skewed, but were corrected with a reflected 1.25 root transformation (Osborne, 2010). Table 5 demonstrates that while several items showed significant differences by EHS Version (*N* = 886), Child Age in days (*N* = 886), and Country (UK, *N* = 674, vs. USA, *N* = 112; UK vs. Australia, *N* = 30), all Zumbo–Thomas scores were below .075. This suggests there were no meaningful item differences for EHS Version, Child Age in days, Parent Education, or Country (UK vs. USA; UK vs. Australia). There were no significant differences for Parent Education (with degree, *N* = 730; without degree, *N* = 141).

**Table 5:** Differential item functioning for EHS Version (Preliminary, Final), Child Age, Parent Education, and Country. ΔR^2^ are Zumbo–Thomas effect sizes. Significant *p* values are .0025 for EHS Version and Child Age, to account for Bonferroni corrections. Significant *p* values are .05 for Parent Education and Countries to account due to smaller sample sizes (Belzak, 2020). *NA* = Not Applicable, as *p* values were not significant

**Items**	**Humor type**	**EHS version**		**Child age**		**Parent education**		**UK vs. US**		**UK vs. Australia**	
		***p***	**Δ*R***^***2***^	***p***	**Δ*R***^***2***^	***p***	**Δ*R***^***2***^	***p***	**Δ*R***^***2***^	***p***	**Δ*R***^***2***^
9	Hide & Reveal Games	0.157	*NA*	**< .001**	**0.05**	0.069	*NA*	**0.001**	**0.054**	0.653	*NA*
8	Tickling	**< .001**	**0.041**	**< .001**	**0.064**	0.862	*NA*	0.411	*NA*	**0.035**	**0.019**
17	Funny faces	0.209	*NA*	**< .001**	**0.058**	0.941	*NA*	0.755	*NA*	0.997	*NA*
10	Bodily humor	0.015	*NA*	**< .001**	**0.044**	0.504	*NA*	0.795	*NA*	0.428	*NA*
1	Funny voices	0.063	*NA*	0.003	*NA*	0.638	*NA*	0.312	*NA*	0.547	*NA*
12	Chasing	**< .001**	**0.031**	**< .001**	**0.028**	0.171	*NA*	**0.01**	**0.013**	**0.025**	**0.012**
3	Misusing objects	**0.001**	**0.017**	**< .001**	**0.023**	0.532	*NA*	0.927	*NA*	**0.047**	**0.01**
19	Teasing	0.144	*NA*	**< .001**	**0.038**	0.301	*NA*	0.244	*NA*	0.473	*NA*
18	Showing hidden body parts	0.086	*NA*	**< .001**	**0.02**	0.569	*NA*	**0.032**	**0.009**	0.109	*NA*
11	Scaring others	0.141	*NA*	**< .001**	**0.026**	0.635	*NA*	**0.006**	**0.013**	0.121	*NA*
15	Acting like something else	0.203	*NA*	0.908	*NA*	0.110	*NA*	0.801	*NA*	0.613	*NA*
5	Taboo topics	0.011	*NA*	0.003	*NA*	0.632	*NA*	0.457	*NA*	0.226	*NA*
6	Mislabeling	0.685	*NA*	**< .001**	**0.037**	0.150	*NA*	**0.028**	**0.006**	0.875	*NA*
2	Making fun	0.003	*NA*	**< .001**	**0.041**	0.388	*NA*	0.284	*NA*	**0.001**	**0.013**
7	Aggressive humor	0.007	*NA*	**< .001**	**0.04**	0.775	*NA*	0.696	*NA*	0.808	*NA*
4	Playing with concepts	0.859	*NA*	**< .001**	**0.05**	0.201	*NA*	**0.001**	**0.011**	0.66	*NA*
16	Nonsense words	0.018	*NA*	**< .001**	**0.018**	0.706	*NA*	**< .001**	**0.017**	0.093	*NA*
13	Playing with social rules	0.157	*NA*	0.679	*NA*	0.389	*NA*	0.224	*NA*	0.653	*NA*
14	Tricks	**< .001**	**0.033**	0.439	*NA*	0.709	*NA*	**0.043**	**0.012**	0.178	*NA*
20	Puns	**< .001**	**0.074**	0.185	*NA*	0.503	*NA*	**< .001**	**0.055**	0.182	*NA*

We then examined internal reliability for each year of Child Age; each level of parent education; and within each country (UK, USA, Australia). The EHS’s internal reliability was good in children under 1 year (*N* = 126, *KR20* = 0.83), 1-year-olds (*N* = 293, *KR20* = 0.73), 2-year-olds (*N* = 269, *KR20* = 0.76), and 3-year-olds (*N* = 198, *KR20* = 0.75). The EHS’s internal reliability was very good for both Parent Education categories: participants who had a university degree (*N* = 730, *KR20* = 0.89), and parents who did not have a university degree (*N* = 141, *KR20* = 0.87). The EHS’s internal reliability was very good for participants in Australia (*N* = 30, *KR20* = 0.91), the United Kingdom (*N* = 674, *KR20* = 0.87), and the United States (*N* = 112, *KR20* = 0.88).

#### Age of emergence

**Fig. 4:**
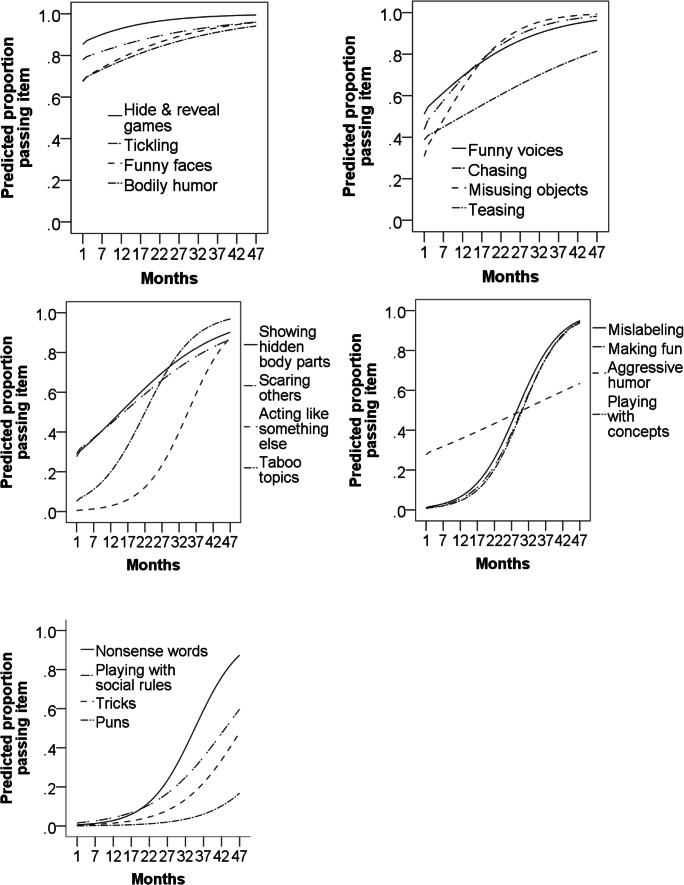
Age curves for each item. Items are grouped in the order of age of emergence by percentiles (see Table 1). Participants included all children from Studies 2 and 3, *N* = 671

To get an idea of when each type of humor emerges, we combined all data from Studies 2 and 3, where children were over 1 month (*N* = 671) and ran binary logistic regressions with each EHS item as the dependent variable, and age in months as the independent variable. Age was a significant predictor of each item, all Wald > 11.77, *β* > .032, *p* < .002. We then plotted the predicted proportion of children passing each item, by age (see Figure 4). Table 1 summarizes the ages at which 25, 50, and 75% of children pass each item. Visual inspection of Fig. 4 suggests that some items group by age trajectories. For instance, tickling, hide and reveal games, funny faces, and bodily humor appear to group together, and these may all capture body-based humor. Teasing, showing hidden body parts, and scaring others group together, and may capture something akin to “naughtiness.” Acting as something else, taboo topics, mislabeling, making fun, playing with concepts, and nonsense words group together, and may all require representational understanding, including language. Playing with social rules and tricks group together, and may both require an advance level of social cognition. Finally, Funny voices, chasing, and misusing objects group together, but it is not clear what they have in common.

**Fig. 5:**
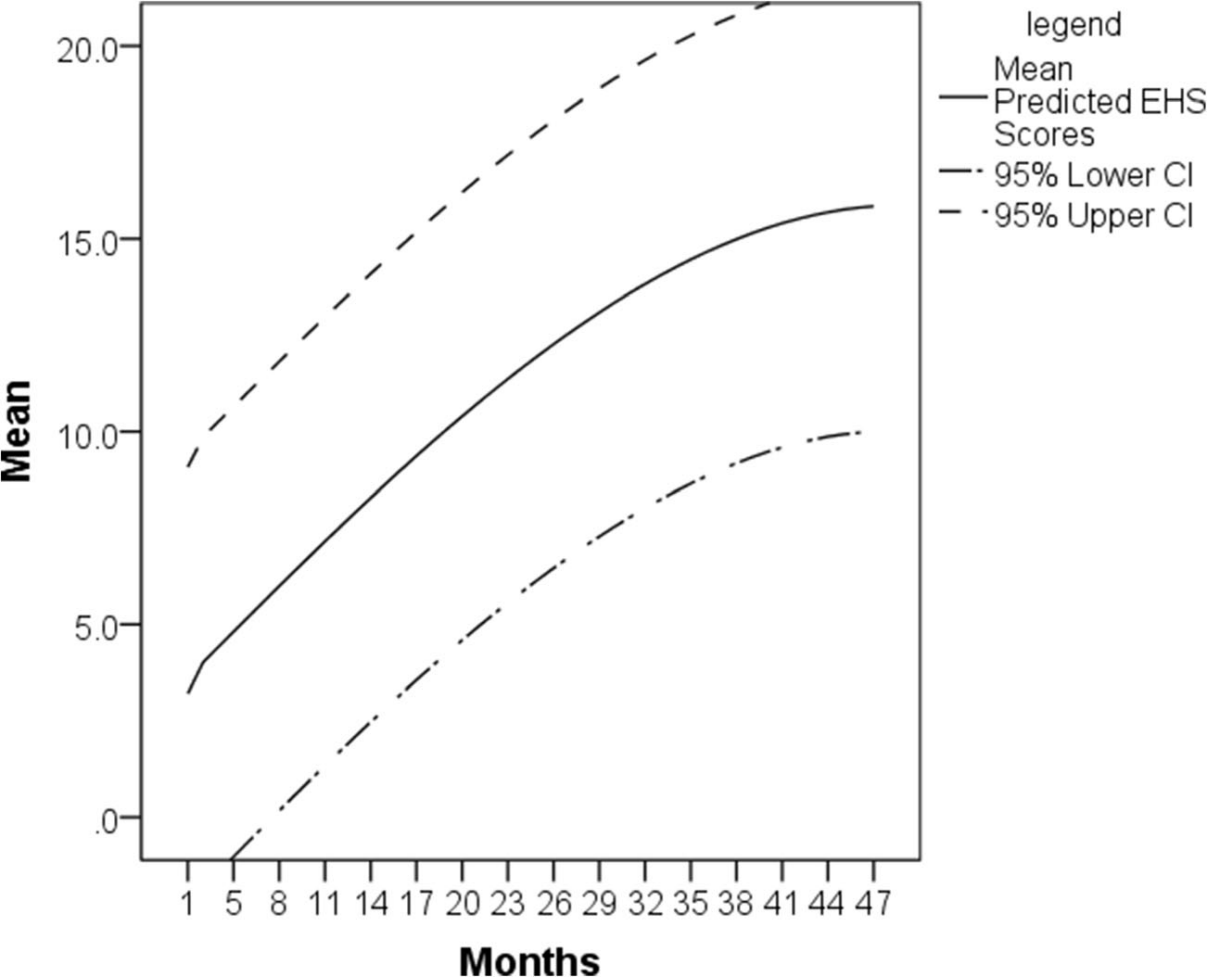
Predicted EHS scores by month, with 95% confidence intervals (CI). While the lower CI is below 0 at 7 months, and the upper CI is above 20 from 34 months, we limited the graph to the range of scores possible on the EHS

To give us a picture of overall expected humor development by age, we ran a stepwise linear regression on the total EHS score as the dependent variable, and age in months, age in months squared, and age in months cubed, as the independent variables. The model, *N* = 671, *F*(2, 668) = 374.16, *p* < .001, found age in months, *β* = 1.071, *t* = 14.34, *p* < .001, and age in months squared, *β* = – .382, *t* = – 5.12, *p* < .001, both predicted the EHS, while age in months cubed did not improve the model fit. We then plotted the predicted EHS scores of children, by age, as well as 95% confidence intervals (see Fig. 5). Figure 5 demonstrates that by 8 months we are 95% confident that the mean score is above 0.

#### Demographic differences

We pooled data across all three samples (*N* = 886) to determine which factors correlated with EHS scores with small to large effect sizes, depending on the sample size (see Table 2). EHS scores were negatively skewed, but were corrected with a reflected 1.25 root transformation (Osborne, 2010). Childcare Hours were positively skewed, but were corrected by changing outliers to be within 3 standard deviations of the mean, and using a 1.5 root transformation. Income in both the UK and USA were positively skewed. Outliers were changed to be within 3 standard deviations of the mean for UK income, and then the variable was transformed with a square root transformation. There were no outliers in the USA sample, so income was transformed with a square root transformation only. Since Child Age (months) and the square of Child Age (months) were strong correlates of the EHS, we always included these variables in the models. We also included the Survey Version (Preliminary EHS, or the final version) in the model in case this affected results. We ran ANCOVAs with EHS scores as the dependent variable; Child Age (months), the square of Child Age (months), and Survey Version as covariates; and either Child Gender (small effect size: *N* = 450 female, *N* = 434 male), Parent Gender (large effect size: *N* = 794 female, 51 male), Parent Education (small to medium effect size, with degree, *N* = 730; without degree, *N* = 141) Country (large effect size, Australia *N* = 30, Canada *N* = 16, United Kingdom *N* = 674, United States *N* = 112), Multilingualism (small to medium effect size: *N* = 142 multilingual, *N* = 695 monolingual), or Siblings (small to medium effect size: *N* = 386 with siblings, *N* = 461 without siblings), as the independent variable. None of the ANCOVAs violated Levene’s Test of Equality, all *F* < 2.54, *p* > .113. None of these variables had a significant effect on EHS scores, all *F* < 2.15, *p* > .145. We also ran linear regression models with the EHS scores as the dependent variable; Child Age (months), the square of Child Age (months), and Survey Version as independent variables in step 1; and either Parent Education (small effect size: *N* = 876), Parent Age (small effect size: *N* = 843), or Childcare Hours (small to medium effect size: *N* = 604), as the independent variable in step 2. None of these were significant, all *t* < 0.91, *p* > .363. As different countries have different currencies and levels of income, we examined the United Kingdom (small to medium effects size: *N* = 433) and the United States (medium to large effect size: *N* = 89) for effects of income only due to sample size. We ran linear regression models with the EHS as the dependent variable; child age (months), the square of child age (months), and survey version as independent variables in step 1; and income (transformed) as the independent variable in step 2. Income was not significant for either country, both *t* < 1.68, *p* > .097.

### Discussion

The EHS did not show any differences in item functioning across survey version, child age, parent education, or country (UK vs. USA; UK vs. Australia). This suggests the EHS could be used across these demographic groups. However, caution should be taken with the results for education and country as we could only look at item functioning differences for medium or large effect sizes. Future research should examine whether differences exist when powering for smaller effect sizes between education levels or countries. Additionally, there was good internal reliability across child age groups (by year), parent education level (degree, no degree), and country (UK, USA, Australia).

The only demographic difference we identified for the EHS was age, and this was shown for each EHS item as well. The binary logistic regressions for each item could be useful for parents, early years educators, and professionals working in children’s media, in identifying which types of humor to target for different age groups.

For some demographic variables, we had enough power to rule out even small effect sizes, including child gender and parent age. Therefore, we can be fairly confident that the EHS shows no mean differences across these demographic variables. While there were no other significant EHS mean differences related to demographic variables, these were powered for small to medium, up through large, effect sizes. Therefore, future research should target specific samples, e.g., fathers, multilingual children, etc., to examine whether any small effect size differences exist.

## General discussion

This study found the 20-question EHS is for the most part a reliable measure of humor development from 1 to 47 months. The survey showed high internal reliability across separate groups of parents, and this extended to parents from different countries (Australia, United Kingdom, United States), different educational backgrounds (with and without degrees), and for different ages groups (0–3 years). The survey also showed good inter-rater reliability between parents, and good longitudinal stability at 6 months’ time. While the scores between the EHS and the humor experiment in the lab showed an initial correlation, this disappeared when age was controlled for. This suggests that while the EHS is reliable in terms of parental inter-observability, lab experiments do not necessarily capture the everyday humor reported by parents.

This is the first study demonstrating a comprehensive taxonomy and pattern of development of humor in the first four years of life. This builds on previous research demonstrating that a variety of types of humor are appreciated in the first year (Reddy, 2001; Sroufe & Wunsch, 1972), and beyond (Hoicka & Akhtar, 2012; Johnson & Mervis, 1997; Loizou, 2005), but brings it all together to gain a comprehensive view of how humor emerges and builds. These findings are important as they can be used for future humor research, ensuring that experiments, observations, and parent reports are based on documented types of humor, and allow researchers to focus in on appropriate types of humor for their study’s age range. This information is also useful for early years educators, parents, and children’s media, who can use this information to plan lessons, bond with their children, and create successful books, television shows, and apps for their target audiences, respectively. However, while fairly comprehensive, it is still possible that we have missed out on some common types of humor in the first 4 years. For instance, while irony is not typically understood until at least 4 years following experimental evidence (Angeleri & Airenti, 2014), there are reports that some children understand it as early as 2 years (Airenti, 2016). Future research should further examine the scope of humor in the early years, and also perhaps broaden the taxonomy beyond 3-year-olds.

The EHS is an important tool as it will allow us to efficiently determine how humor emerges in the first place. The survey, which takes less than 5 minutes to complete, could be combined with other developmental surveys, covering, for instance, language, motor skills, cognition, and social cognition (Baker et al., 2013; Hoicka et al., 2021; Fenson et al., 1994; Libertus & Landa, 2013; Tahiroglu et al., 2014) to understand humor’s origins. Different theories have suggested humor development is cognitive, social, or socio-cognitive in nature (Freud, 1916; Loizou, 2005; McGhee, 1979; Shultz, 1976), and the EHS could help us more easily determine which of these theories are best supported (if not all of these). Furthermore, as humor is linked to coping with stress, making friends, learning, and being creative (Bressler et al., 2006; Hoicka & Martin, 2016; Martin & Dobbin, 1988; Wanzer et al., 1996; Ziv, 1983), the EHS provides a tool to more easily examine these potential relationships in early development.

Our results based on demographics found that, unsurprisingly, older children had higher humor scores. Yet our age findings are useful as they give us an initial idea, based on a sample of almost 700 participants, of what typical humor development is. In the future, with even larger samples, the EHS may be able to serve as a diagnostic tool for developmental differences, e.g., autism spectrum disorder, which shows early developmental differences in humor (Baron-Cohen, 1997; Reddy et al., 2002).

No other demographic differences were found. This suggests humor may develop similarly across boys and girls; English-speaking countries; varying socio-economic statuses (parents’ education level; and household income, within the UK and USA only); and varying social environments, i.e., having siblings or not, and amount of time spent in childcare. However, caution should be taken in these results as only child gender and parent age were powered for a small effect size. Therefore, future research should examine whether there are small differences for these demographic variables.

The main concern with the EHS is the lack of concurrent validity with the lab study. One possibility is that parents are not good at reporting their children’s behaviors. However, past research shows this is not the case, as there is good inter-observer reliability between parent reports and lab tasks in the early years for cognition (Baker et al., 2013), social cognition (Hoicka et al., 2021; Hutchins et al., 2012; Tahiroglu et al., 2014), motor skills (Libertus & Landa, 2013), as well as parents’ own parenting styles towards their children, when it comes to support (Winstanley & Gattis, 2013). A second possibility is that parents are not good at reporting humor in particular. However, this seems unlikely as jokes would appear more tangible to report on than any of the other above-reported skills. A third possibility is that the lab task did not adequately capture humor. However, past research indicates infants and toddlers show an understanding of humor in the lab (Hoicka & Akhtar, 2011; Hoicka et al., 2017; Hoicka & Gattis, 2008; Hoicka & Wang, 2011; Mireault et al., 2014; Mireault et al., 2015). Furthermore, we found that children laughed, and reproduced acts while smiling or laughing, more during joke trials than control trials, suggesting it worked well as a humor experiment at the group level, but perhaps not on an individual differences level. One possible problem with our study was our stop-rule. We stopped the experiment early if children did not laugh or produce any of the jokes in a block, to avoid stress for young participants. However, this will have also limited our ability to observe children’s responses to all types of humor.

Relatedly, Mireault, Sparrow, et al. (2012) found no correlation between parent reports of 6-month-olds’ smiling and laughter, and researcher’s observations of smiling and laughter during a 10-minute video in which parents tried to make their infants laugh. They drew on theory from Ruch et al. (1996) suggesting state and trait humor are related, but not the same thing. While trait humor is a necessary condition for state humor, it is not sufficient (Ruch et al., 1996). Indeed, the humor appreciation and production scores in our lab study were much lower than the EHS scores suggesting this is the case. Therefore, our lab task may not have had sufficient conditions to translate children’s natural day-to-day trait humor into state humor during the task. One sufficient condition that might not have been captured in our lab task is that while children might appreciate some specific joke tokens of a certain joke type, this does not mean they will appreciate all joke tokens of a certain joke type. For instance, we asked parents if their child has ever appreciated or produced a joke involving “Strange actions with objects, e.g., use wrong end of spoon, put cup on head.” Many jokes could fulfil the requirements to answer positively to this question – a child could find any *one* of the following funny: a spoon on one’s nose; sitting on a phone; putting a sock in one’s mouth; sitting upside down on a chair; etc. However, in the experiment, they had only one specific joke token they could appreciate to pass this item: children had to find it funny that the experimenter put a boot on her hand. Therefore, while some children may have appreciated other joke tokens of this type, if they did not appreciate this particular joke token, they would not score a point. This could lead to variation in the lab scores, making it more difficult to get a correlation with the EHS.

Another possible factor is that an experimenter performed all the jokes. While the experimenter did warm up with the child beforehand, she was a new person, and this may have made it more difficult for some children to show humor appreciation. For instance, infants are more likely to laugh when a parent plays peekaboo with them, but more like to cry when a stranger does so (MacDonald & Silverman, 1978). While some children may have had no problems joking with a new person, other children may have been shy, or not had the common ground to appreciate jokes with them, leading to more variation in our lab results. One possibility would be to, in future, control for temperament when running humor experiments. Indeed, temperament traits lead to differences in humor processing and laughter in lab situations for older children (6–13 years) and adults (Mobbs et al., 2005; Ruch, 1994; Ruch & Deckers, 1993; Samson et al., 2009; Vrticka et al., 2013).

### Limitations and future directions

One limitation of the EHS is that the main instructions may be confusing. We asked parents, “For the following, tick Yes if your child finds it funny when others make this joke type and/or makes this joke type him/herself to be funny.” This is a long sentence with several clauses, using two slash signs. This might be better worded, e.g., “For the following, tick Yes if your child finds these types of jokes funny.” Furthermore, the EHS was used across different countries, however item content and wording may need to be different for different countries. For instance, one item was “Socially unacceptable situations, e.g., putting cat on dining table, saying naughty words, etc.” While the word “naughty” would be fairly normal in a British population, this word might seem a bit out of place in a North American context. Parent interviews should be used across different countries in future to determine whether parents understand the instructions and the items (DeVellis, 2017). Relatedly, there are cultural differences in humor across English-speaking countries, e.g., American adults report using more social humor than Brits, and Brits have a more negative attitude towards humorous people than Australians (Martin & Sullivan, 2013). Therefore, parent interviews might also better help understand how items might be viewed differently across cultures. Additionally, while our DIF analyses suggested no differences in how parents responded to items by country, the analyses were not powered for a small effect size. Future research should power for a small effect size.

A second limitation involves sampling. Twenty-four children chose not to participate in our lab task in Study 3, therefore we may have excluded children who were, e.g., more shy. Our sample in Study 3 may have therefore been self-selected, and thus unrepresentative of children more generally.

A third limitation is that Study 1 demonstrated that humor production and appreciation are difficult to distinguish. While humor appreciation rates were generally higher than production rates, the two were highly correlated. The EHS cannot, therefore, be easily used for studies interested *only* in either humor appreciation or production.

A final limitation is that, while our CFA suggested items primarily grouped onto one factor, our logistic regression analyses by age (see Fig. 4), suggests that some items group by age trajectories. For instance, tickling, hide and reveal games, funny faces, and bodily humor appear to group together, and these may all capture body-based humor. This may mean that, when comparing the EHS to other factors (e.g., motor control, language, and social cognition), some factors may load more strongly onto some sets of items than others. It may, therefore, be useful to consider grouping items within the EHS when the research question involves e.g., motor control, language, or social cognition.

### Conclusions

The EHS shows us for the first time the taxonomy of humor development in the first years of life. As well as giving us a much fuller picture of how humor develops, the EHS offers an efficient tool to further examine the origins of humor (e.g., cognitive and social development), as well as how humor may affect other aspects of life (e.g., coping with stress, creativity) in early life. Finally, the EHS has the potential, with more research, to be used as a diagnostic tool in early development in terms of developmental differences.

## Unpublished parent reports of humor involving scaring others and playing tricks from the Hoicka & Akhtar (2012) corpus.

**Scaring:**

“Scaring us as he is a lion/soldier etc.”

“He has hidden in my room under a blanket and waited so patiently for up to 5 minutes for me to come in and be ‘missing.’ We've definitely hidden under blankets, but never for that long and never waiting to make a joke by surprising someone.”

“Hides behind couch and jumps up saying 'boo'. He finds this so funny. But recently instead of just standing straight up he peeks his head round the side to surprise you, and looks very pleased with himself!”

**Tricks:**

“He says thing are [a] different color then they are to trick us.”

“Tries tricking us with animal noises ... saying that we get them wrong and she’s right then says we are silly because they do make them noises.”

**Table 6:** Detailed breakdown of ethnicity of children and parents across studies, as well as income and recruitment information

	**Study 1**	**Study 2**	**Study 3**
**No. of children/ parents**	219	587	84
**Children’s ethnicity:**			
Black	2 (1%)	6 (1%)	0 (0%)
East Asian	1 (0.5%)	3 (0.5%)	0 (0%)
Hispanic	1 (0.5%)	0 (0%)	0 (0%)
Pacific Islander	0 (0%)	1 (0.2%)	0 (0%)
South Asian	3 (1%)	6 (1%)	0 (0%)
West Indian	0 (0%)	1 (0.2%)	0 (0%)
White	186 (85%)	531 (90%)	78 (93%)
Of Mixed Ethnicity	8 (4%)	17 (3%)	5 (6%)
Other (not specified)	12 (5%)	17 (3%)	0 (0%)
Not reported	6 (3%)	5 (1%)	1 (1%)
**Parents’ ethnicity:**			
**Black**	2 (1%)	7 (1%)	1 (1%)
**East Asian**	4 (2%)	8 (1%)	0 (0%)
**South Asian**	3 (1%)	5 (1%)	0 (0%)
**West Indian**	0 (0%)	1 (0.2%)	0 (0%)
**White**	201 (92%)	512 (87%)	73 (87%)
**Of mixed ethnicity**	0 (0%)	8 (1%)	2 (2%)
**Other (not specified)**	5 (2%)	14 (2%)	0 (0%)
**Not reported**	4 (2%)	32 (5%)	8 (10%)
**Household income**			
Australia: *N* *Median* *Range* *SD*	18 $122,500 AUD $40,000–$240,000 $55,020	*NA*	*NA*
Canada: *N*	7	5	*NA*
*Median* *Range* *SD*	$150,000 CAD $60,000–200,000 $49,051	$100,000 $50,000–160,000 $40,927	
United Kingdom: *N* *Median* *Range* *SD*	76 £54,000 GBP £13,000–£150,000 £28,462	285 £50,000 £6,000–£750,000 £51,713	75 £53,000 £12,000–£120,000 £23,051
United States of America:			
*N*	36	56	*NA*
*Median*	$154,000 USD	$125,000	
*Range*	$15,000–$350,000	$20,000–$250,000	
*SD*	$74,119	$61,042	
**Recruited**			
www.babylovesscience.com	219 (100%)	455 (78%)	0 (0%)
University of Sheffield Cognitive Development Lab	0 (0%)	123 (21%)	84 (100%)
PASE Early Years Labs	0 (0%)	9 (2%)	0 (0%)

**Table 7:** Questions for the Preliminary EHS and the EHS. Question block 1 was used in the Preliminary EHS only. Question block 2 was used in both the Preliminary EHS and the EHS. The Preliminary EHS used incremental questioning, while the EHS used Yes/No questions. Questions with letters are contingent on their route items, e.g., question 2a is asked only if the parent responds “Yes” to question 2. Questions with Roman numerals are contingent on their route items, e.g., question 12a(i) is asked only if the parent responds “Yes” to question 12a.

	**General humor questions**
1	Does your child laugh?
2	Does your child appreciate humor? (It could be verbal or physical, e.g., silly faces)
2a	When was the last time your child appreciated humor?
3	Does your child intentionally produce humor (could be physical or verbal, e.g., silly faces; you might not get the humor)
3a	When did your child last intentionally produce humor? (It may or may not have been humorous to you).
	**Specific joke questions**
Instructions - Preliminary EHS	Types of Jokes: We will now ask questions about specific types of jokes your child may enjoy. When we ask if your child copies jokes, we mean making the exact same joke (or at least trying to). For instance, if they put a sock on their head after watching someone else do so. When we ask if your child invents jokes, we mean do they make jokes that (as far as you know) they have never seen anyone else do. Please choose the answers that best describe your child.
Preliminary EHS Question Structure*:	
a	Has your child ever seen anyone make this type of joke?
a(i)	Has your child ever found it funny when others produced this type of joke?
b**	Has your child ever tried to make this type of joke?
b(i)	Has your child ever correctly copied this type of joke from others?
b(ii)	Has your child ever invented this type of joke correctly him/herself?
Instructions - EHS	For the following, tick Yes if your child finds it funny when others make this joke type and/or makes this joke type him/herself to be funny.
J1	Making strange voices (not just strange noises)
J2	Making fun of others, e.g., calling someone a poopoohead
J3	Strange actions with objects, e.g., use wrong end of spoon, put cup on head
J4	Saying strange things/mixing up concepts/nonsense (e.g., dinosaurs eat the wall; cats have 5 legs, dogs say moo), including nonsense variations of knock-knock/why did the chicken cross the road jokes
J5	Referring to gross things, e.g., poo, sneezing, smelly feet, etc.
J6	Mislabeling objects/events, e.g., calling a car a banana; could be in song, or intentionally giving you the wrong answer
J7	Aggressive acts, e.g., spitting out water, throwing things, pushing people, etc.
J8	Tickling, including variations, e.g., using objects to tickle, e.g., stick or feather
J9	Peekaboo/ hide & seek, including variations, e.g., hiding objects in bags and revealing them
J10	Strange body movements, e.g., head through legs, kicking legs in air
J11	Scaring people, e.g., jumping out at them, or yelling
J12	Chasing, including variations, e.g., making toys chase each other
J13	Socially unacceptable situations, e.g., putting cat on dining table, saying naughty words, etc.
J14	Playing tricks on people, e.g., putting salt in the sugar bowl
J15	Acting like something else, e.g., an animal, another person, etc.
J16	Inventing words, e.g., schmoogly
J17	Pulling/making silly faces, e.g., scrunching up face
J18	Showing normally hidden body parts, e.g., lifting shirt to reveal tummy; taking off clothes
J19	Teasing, e.g., offering an object and taking it away
J20	Making puns, that is, jokes where words have double meanings, e.g., Why are fish so smart? Because they live in schools
J21^ʈ^	Making strange noises, e.g., raspberries, shrieks, sneeze sounds

*Question structure used for questions J1–J21 of the Preliminary EHS. **Contingent on answering “Yes” to question 3 of the General Humor Questions. ^ʈ^Question included in the Preliminary EHS only.

**Table 8:** Actions for the lab experiment

Humor type	Joke	Control	Materials
Funny noises	Makes a monkey squawk three times	Hums, ‘twinkle twinkle little star’ tune to child	Toy monkey
Peekaboo	Quickly hides face behind hands and shows face, saying, “boo!”	Waves at child	*NA*
Tickling	Tickles teddy bear’s tummy and says, ‘tickle, tickle, tickle!’	Cuddles teddy bear	Teddy bear
Funny faces	Pulls mouth to sides with fingers and sticks out tongue	Scratches face	*NA*
Bodily humor	Humorously waggles arms	Claps hands	*NA*
Misusing objects	Puts glove on foot	Puts glove on hand	Glove
Chasing	Makes a toy pig chase a toy cow and says, “I’m gonna get you!”	Makes a toy pig and toy cow walk side by side and says, “We’re going for a walk!”	Toy pig, toy cow
Funny voices	Speaks in a humorous high voice to say, “The dog is crossing the road”	Uses a normal voice to say, “The dog is crossing the road”	Toy dog
Acting like something else	Gets down on all fours, mimicking a dog, and says, ‘Woof! Woof!’	Walks around the room and says, “I like walking!”	*NA*
Teasing	Teases by offering and withdrawing a feather ball toy from parent	Offers to give feather ball toy to parent. Lets parent take the toy.	Feather ball toy
Scaring others	Yells, “boo!” at parent while their back is turned. Parent reacts scared	Says, “Hello!” to parent	*NA*
Showing body parts	Lifts top of doll and shows stomach, says, ‘Look! Her tummy!’	Covers doll over with small towel and says, ‘Look, a blanket!’	Doll, doll top, small towel
Taboo topics	Smells the doll’s bum and says, “Ewww! It’s smelly!”	Holds doll out in front of them, looks at doll and says, “I like this doll!”	Doll
Mislabeling	Holds a hat and says, “This is a sheep!”	Holds a toy sheep and says, “This is a sheep!”	Hat, toy sheep
Aggressive humor	Parent builds a tower using toy blocks, and then the experimenter knocks it over. Parent looks surprised	Builds a tower using blocks	Blocks
Making fun	Drops straws on parent’s head. Parent looks surprised	Puts a hat on the parent’s head	Straws, hat
Playing with concepts	Holds a toy horse and says, “The horse goes Quack! Quack Quack!”	Holds a toy horse and says, “The horse goes neigh! Neigh!”	Toy horse
Nonsense words	Holds a spoon and says, “This is a schmoogly”	Holds a spoon and says, “This is a spoon”	Spoon
Playing with social rules	Leans back and puts feet on table	Holds a book and puts it on the table	Table, book
Tricks	Says to parent, “I’ve got you a nice gift!” Hands gift to parent, waits for parent to open gift to reveal crumpled paper inside. Parent looks disappointed.	Says to parent, “I’ve got you a nice gift!” E hands gift to parent, waits for parent to open gift to reveal a toy plane inside. Parent looks happy.	Crumpled paper, toy plane
Puns	Says, “Why are teddy bears never hungry? Because they’re always stuffed!”	Says, “Why are teddy bears never hungry? Because they eat a lot!”	*NA*
